# Optical Quality of the Cornea After Orthokeratology Based on Total Corneal Refractive Power, Corneal Asphericity, and Epithelial Thickness: Protocol for a Prospective Quasi-Experimental Pre-Post Study

**DOI:** 10.2196/94476

**Published:** 2026-08-12

**Authors:** José Licari, Alba Medico-Salsench, Laura Medina-Guijarro, César Villa-Collar, Cristina Rey-Reñones

**Affiliations:** 1 Clinica Licari, Tarragona, Tarragona, Spain; 2 Department of Basic Medical Sciences, Faculty of Medicine and Health Sciences, Universitat Rovira i Virgili, c/ Sant Llorenç, 21, Reus, Tarragona, Spain, 34 977759334 ext 9334; 3 Faculty of Biomedical and Health Sciences, Universidad Europea de Madrid, Madrid, Spain

**Keywords:** myopia, astigmatism, corneal wavefront aberration, visual acuity, retina, optometrists

## Abstract

**Background:**

Orthokeratology is a nonsurgical corneal reshaping technique used for the temporary correction of myopia and for myopia control. Nighttime use induces central epithelial thinning and midperipheral thickening, altering corneal asphericity and higher-order aberrations.

**Objective:**

The primary objective is to determine the change in anterior corneal spherical aberration after 1 month of orthokeratology use. Secondary objectives include evaluating changes in total corneal refractive power, the Q value, and epithelial thickness.

**Methods:**

This prospective quasi-experimental pre-post study was conducted at a private ophthalmology clinic (Licari Vision Clinic, Tarragona, Spain). Participants were patients aged 8 to 21 years with myopia ranging from −1.00 to −5.00 D who underwent nighttime orthokeratology for 1 month. Outcome measurements included Pentacam tomography, anterior segment optical coherence tomography (OCT), and Zernike analysis, all performed in the morning 1 to 3 hours after lens removal. Cycloplegic refraction was performed at baseline only to confirm eligibility criteria and inform orthokeratology lens design. A minimum of 23 participants was required to detect a change of ≥0.14 μm in anterior corneal spherical aberration (α=.05; β=.05; power=95%; SD 0.166 μm), incorporating a 20% dropout rate. The unit of analysis will be the eye, including both eyes of each participant. To account for the nonindependence of contralateral eyes in the same participant’s within-subject repeated measurements, the primary and secondary outcomes will be analyzed using linear mixed-effects models with a fixed effect for time (baseline vs 1 month) and a random intercept for each participant. A significance level of *P*<.05 will be applied in all cases.

**Results:**

The protocol received a favorable ethics opinion from the Drug Research Ethics Committee (*Comité de Ética de la Investigación con medicamentos* [CEIm]) of the Pere Virgili Institute for Health Research (*Institut d'Investigació Sanitària Pere Virgili* [IISPV]) in October 2024 (CEIm Code 159/2024) and was registered at ClinicalTrials.gov (NCT06775509). This study received no external funding. Recruitment was completed. Data collection has been finalized, and data analysis is currently underway. The study findings are expected to be published in January 2027.

**Conclusions:**

Corneal optical quality may be modified after 1 month of orthokeratology lens use, and the findings may contribute to refining clinical decision-making regarding the use of orthokeratology for myopia management.

## Introduction

The cornea is the eye’s transparent, avascular tissue, comprising 5 distinct layers: the corneal epithelium, Bowman membrane, the stroma, Descemet membrane, and the endothelium. Its thickness varies, being thinner centrally (approximately 0.52 mm) and reaching nearly 1 mm near the limbus. The outermost layer, the corneal epithelium, is vital not only for protection but also for making a significant contribution to corneal refractive power.

The human cornea is naturally prolate, with a steeper center and a flatter periphery, reflected by a mean Q value of approximately −0.27. This asphericity minimizes spherical aberration under physiological conditions; in contrast, oblate corneas (Q>0), which are steeper in the periphery, exacerbate spherical aberrations.

Orthokeratology is a clinical technique that temporarily reshapes the cornea to eliminate or reduce refractive error [1]. Orthokeratology lenses are typically worn overnight (overnight orthokeratology) and removed upon waking, allowing patients to achieve clear vision without correction throughout the day. These lenses are made of rigid gas-permeable (RGP) material and are designed to reduce myopia by flattening the central cornea [2]. This treatment is reversible, safe, and approved by the US Food and Drug Administration (FDA). Although myopia correction and control are the most widely studied applications of orthokeratology, lens designs for the correction of hyperopia and astigmatism have also been developed and reported in the literature, broadening the clinical scope of this technique.

Although orthokeratology effectively reduces myopia [3] and enables good uncorrected vision during the day, numerous studies have demonstrated that the combination of central corneal flattening and midperipheral corneal thickening leads to a significant increase in higher-order aberrations (HOAs), resulting in decreased visual quality [4,5].

With the use of orthokeratology, higher-order corneal aberrations increase significantly, particularly spherical aberration [6], and these increases correlate with the degree of myopic correction [7,8]. Several studies have concluded that the loss of optical quality is most evident within the first month but is not significant in subsequent months. Following short-term corneal remodeling, optical quality decreases to some extent, but the loss is considered acceptable [9-11].

As previously mentioned, orthokeratology causes central corneal epithelial thinning and midperipheral epithelial thickening [12]. Changes in epithelial thickness exhibit a nonuniform pattern, with greater thinning in the temporal and inferior regions than in the nasal and superior regions of the paracentral area and more pronounced thickening in the nasal region than in the temporal region of the midperiphery [12,13].

Despite the growing body of evidence on the optical effects of orthokeratology, previous studies have consistently reported that the optical deterioration induced by orthokeratology is fully established after 1 month of treatment, with no significant further changes observed thereafter. For this reason, the 1-month time point was selected to capture the maximum expected optical deterioration, as described in the literature. However, most of the previous evidence has been derived from analyses limited to the anterior corneal surface. This study aims to confirm whether these previously reported changes are also present when the whole cornea is systematically evaluated, including total corneal refractive power (TCRP) and total corneal aberrations derived from both the anterior and posterior surfaces, and to confirm the expected epithelial redistribution pattern, characterized by central thinning and midperipheral thickening. Accordingly, this study aims to determine the change in anterior corneal spherical aberration after 1 month of overnight orthokeratology use as its primary objective, with secondary objectives including the evaluation of changes in TCRP, Q value, and epithelial thickness.

## Methods

### Study Objectives

The primary objective of this study is to determine the change in anterior corneal spherical aberration, defined as the Zernike coefficient z(4,0) measured at a 7-mm pupil diameter, after 1 month of overnight orthokeratology.

The secondary objectives are to analyze changes in refractive maps obtained using the Pentacam topographic system (OCULUS Optikgeräte GmbH), including TCRP and corneal asphericity; to assess corneal aberrations through Zernike analysis at the remaining pupil diameters, examining the relationship between changes in anterior corneal asphericity and induced spherical aberration; and to evaluate changes in central and peripheral epithelial thickness using corneal optical coherence tomography (OCT) and their correlation with changes in corneal asphericity and induced aberrations.

For clarity, the primary outcome of the study is the change in anterior corneal spherical aberration z(4,0) at a 7-mm pupil diameter, from baseline to the 1-month follow-up visit. All remaining measures constitute secondary outcomes, namely, anterior corneal spherical aberration z(4,0) at 3-mm and 5-mm pupil diameters; spherical-like, coma, coma-like, third-order root mean square (RMS), fourth-order RMS, and total HOA RMS at all 3 pupil diameters; TCRP at 3, 5, and 7 mm; corneal asphericity (Q value); and central and peripheral epithelial thickness. Axial length is reported for descriptive purposes only and does not constitute an outcome measure.

Cycloplegic refraction was performed at the baseline visit only, to confirm the degree of myopia for eligibility assessment and, together with corneal topography, to inform the orthokeratology lens design. It was not repeated at follow-up visits and was not used as a correlational variable in the analysis of posttreatment corneal changes.

### Study Design and Setting

This was a prospective, quasi-experimental, pre-post study. Data were collected at baseline and 1 month after the commencement of the intervention. The study was conducted at the Licari Vision Clinic in Tarragona, Spain. Potential participants and their guardians were fully informed of the study procedures, and informed consent or assent was obtained prior to screening.

### Participant Selection

Textbox 1 shows the inclusion and exclusion criteria for this study.

Textbox 1. **Inclusion and exclusion criteria.**

Lens centration is assessed at the first follow-up visit after the first night of lens wear and serves as a continuation criterion. All other inclusion criteria are assessed during screening.

**Inclusion criteria**

- Age 8 to 21 years and willingness to participate in the study
- Use of orthokeratology lenses for 8 to 10 hours per night after being informed about the risks and benefits of the treatment
- Spherical myopic refractive error between −1.00 and −5.00 diopters (D), with following-the-rule astigmatism of ≤1.50 D
- Best-corrected distance visual acuity of 20/20 or better before treatment
- Orthokeratology lens centration or radial lens decentration of <0.5 mm, as determined by slit-lamp examination

**Exclusion criteria**

- History of rigid contact lens use or ocular or systemic contraindications to orthokeratology identified during routine examination
- Previous refractive modulation procedure of any type
- Corneal pathology, dry eye disease, glaucoma, retinal pathology, strabismus, ptosis, amblyopia, or a history of ocular allergy or infection

### Data Recording and Storage

Upon enrollment, each participant received a study identification number. Baseline examinations were conducted prior to the initiation of orthokeratology lens wear. The baseline protocol comprised objective refraction under cycloplegia, axial length measurement, comprehensive topographic analysis with the Pentacam system, epithelial thickness analysis, pupil diameter measurement, and orthokeratology lens fitting.

Cycloplegic refraction was performed using 1% cyclopentolate hydrochloride (2 drops instilled 5 minutes apart). Refraction was measured 45 minutes after the first drop using a combination of autorefractometry, retinoscopy, and subjective refraction to the maximum plus for maximum visual acuity (MPMVA).

Axial length was measured with the MYAH myopia management system (Topcon) as part of the routine baseline clinical characterization of participants. Axial length data were collected for descriptive purposes only and do not form part of the primary or secondary objectives of the study.

Epithelial thickness was analyzed using the REVO SOCT 27 anterior segment OCT (Optopol Technology; 27,000 A-scans/second). Corneal epithelial thickness maps were obtained automatically using the device’s integrated segmentation software, without manual intervention by the investigator.

Paragon CRT orthokeratology lenses (Paragon Vision Sciences) were used for all participants. The investigator performed lens fitting and evaluated the fit using fluorescein.

At each follow-up visit after the start of orthokeratology lens use, a complete contact lens assessment was performed. All follow-up measurements were performed in the morning, between 1 and 3 hours after lens removal, to ensure consistency across visits and minimize the effect of corneal recovery over time. Each visit comprised slit-lamp examination to evaluate ocular surface integrity and lens fit, visual acuity assessment, corneal topography with the Pentacam system, and epithelial thickness analysis using the REVO SOCT 27 anterior segment OCT (27,000 A-scans/second) to evaluate the corneal response to orthokeratology lens wear. Epithelial thickness maps were obtained automatically using the device’s integrated segmentation software, without manual intervention by the investigator.

Follow-up visits were conducted by the investigator after 1 night, 1 week, and 1 month of lens wear.

### Sample Size

The sample size was determined based on changes in anterior corneal spherical aberration (Zernike coefficient z[4,0], expressed in microns), as it is one of the HOAs most strongly associated with optical quality after orthokeratology. The threshold of 0.14 μm was derived from the Rayleigh criterion (λ/4) and was used as an optical reference value for sample size estimation, rather than as a validated clinical threshold for visual quality. This criterion establishes that an increase in spherical aberration exceeding one quarter of the wavelength of light deteriorates optical quality.

Using a reference wavelength (λ) of 0.555 μm (peak sensitivity of the human visual system), this yields a threshold of 0.555/4≈0.14 μm.

With a 2-sided α of 0.05 (type I error) and a β of 0.05 (type II error), corresponding to a statistical power of 95%, and an assumed SD of 0.166 μm, a minimum of 19 participants was required. Considering a 20% loss to follow-up rate, a total of 23 participants were recruited.

The sample size was calculated using GRANMO (version 8; Municipal Institute of Medical Research) [14].

This calculation was based exclusively on the primary outcome (anterior corneal spherical aberration).

### Description of the Intervention

Participants wore orthokeratology lenses for 8 to 10 hours per night for at least 1 month.

#### Lens Fitting Criteria

Paragon CRT orthokeratology lenses were fitted by the principal investigator following standard orthokeratology fitting procedures. Lens fit was assessed by evaluating the fluorescein pattern under slit-lamp examination to confirm adequate central support, midperipheral alignment, and peripheral clearance. Treatment centration was assessed at each follow-up visit using 2 complementary methods: slit-lamp evaluation of the fluorescein pattern and topographic analysis of the treatment zone using axial and tangential maps obtained with the Pentacam system, which allowed objective verification of the treatment zone centration relative to the corneal apex. Participants exhibiting radial decentration greater than 0.5 mm were excluded from the study.

#### Adherence Monitoring

Compliance with overnight contact lens use was monitored at each follow-up visit using a structured questionnaire, a daily wear diary completed by the participant and their parent or legal guardian, and direct questions asked of the parents at each visit. A minimum wearing time of 8 hours per night was considered necessary; participants who consistently wore contact lenses for less than 8 hours were considered noncompliant and were excluded from the analysis.

#### Discontinuation Criteria

Participants were excluded from the study if any of the following situations occurred: lens decentration greater than 0.5 mm radially, eye infection, lens intolerance, any adverse ocular event, or voluntary withdrawal from the study at any time.

#### Adverse Event Monitoring

Any adverse events occurring during the study period were recorded in the participant’s clinical record and reported to the Drug Research Ethics Committee (*Comité de Ética de la Investigación con medicamentos* [CEIm]) of the Pere Virgili Institute for Health Research (*Institut d'Investigació Sanitària Pere Virgili* [IISPV]) in accordance with applicable regulations. The principal investigator was responsible for identifying, evaluating, and reporting all adverse events.

### Study Variables and Data Sources

#### Personal Variables

These include age (measured in years) and sex (female or male).

#### Ocular Variables

Ocular variables comprise epithelial thickness, corneal power, corneal asphericity, myopia and astigmatism, pupil diameter, topographic changes, and corneal aberrations, defined as follows:

- Epithelial thickness is measured in microns, along with total corneal thickness (pachymetry).
- Corneal power is expressed as TCRP, obtained from the Pentacam tomographic system. TCRP incorporates both the anterior and posterior corneal surfaces together with corneal pachymetry, using the true corneal refractive index of 1.376, thereby providing a more accurate representation of corneal optical power than conventional keratometric indices. TCRP values are extracted for corneal diameters of 3, 5, and 7 mm. Simulated keratometry (Sim-K) values are not used.
- Corneal asphericity is expressed as the Q value, obtained from the Pentacam tomographic system for the anterior corneal surface. A negative Q value denotes a prolate cornea, steeper centrally and flatter peripherally, whereas a positive Q value denotes an oblate cornea.
- Myopia and astigmatism are measured in diopters.
- Pupil diameter is measured under photopic, mesopic, and scotopic conditions (in mm) using the MYAH myopia management system, which incorporates an integrated pupillometer that allows dynamic pupil diameter assessment under standardized luminance conditions.
- Topographic changes, comprising corneal aberrations and corneal refractive power, are measured in the morning, 1 to 3 hours after lens removal, at all time points.
- Higher-order corneal aberrations are analyzed using Zernike coefficients obtained from the Pentacam for pupil diameters of 3, 5, and 7 mm.

The specific aberrations analyzed are the fourth-order spherical aberration z(4,0), expressed in microns; the spherical-like aberration of orders 4 and 6, represented by its RMS and calculated as the square root of the sum of the squares of z(4,0) and z(6,0), expressed in microns; the third-order coma aberration, represented by its RMS and expressed in microns as the square root of the sum of the squares of z(3,−1) and z(1,3); the coma-like aberration of orders 3 and 5, represented by its RMS expressed in microns as the square root of the sum of the squares of z(3,−1), z(1,3), z(5,−1), and z(1,5); the third-order RMS aberration, expressed in microns as the square root of the sum of the squares of z(3,−3), z(3,−1), z(1,3), and z(3,3); the fourth-order RMS aberration, represented in microns as the square root of the sum of the squares of z(4,−4), z(4,−2), z(4,0), z(2,4), and z(4,4); and the total HOA RMS from the third to the sixth order, represented in microns as the square root of the sum of the squares of all Zernike coefficients from orders 3, 4, 5, and 6.

### Statistical Analysis

Quantitative data following a normal distribution will be presented as means (SDs), while nonnormally distributed quantitative data will be expressed as medians (IQRs). The normality of the variables will be assessed using the Kolmogorov-Smirnov test. Categorical data will be reported as frequencies and percentages.

The unit of analysis will be the eye, including both eyes of each participant. To account for the nonindependence of contralateral eyes in the same participant’s within-subject repeated measurements, the primary and secondary outcomes will be analyzed using linear mixed-effects models with a fixed effect for time (baseline vs 1 month) and a random intercept for each participant. Results will be presented as estimated mean differences with 95% CIs. Model assumptions (normality and homoscedasticity of the residuals) will be assessed graphically, and in the event of substantial noncompliance, a generalized estimating equations (GEE) approach with an interchangeable working correlation structure and robust SEs will be used as a sensitivity analysis. Missing data will be treated under the missing at random (MAR) assumption inherent in mixed models, without imputation; the number of eyes and participants providing data at each time point will be reported. Given the presence of multiple secondary outcomes, we will apply the Benjamini-Hochberg false discovery rate procedure to control for multiplicity. A significance level of *P*<.05 will be applied in all cases.

Given the sample size of 23 participants, multivariable regression analysis will not be performed, as the number of observations would be insufficient to support stable and reliable regression models without introducing the risk of overfitting.

The follow-up visits, performed 1 night and 1 week after lens fitting, were conducted solely for the purpose of evaluating clinical safety and adaptation. Measurements of the primary and secondary outcomes were obtained at baseline and at 1 month, constituting a single pre-post comparative period that will be evaluated using the aforementioned mixed-effects models. This manuscript was prepared in accordance with the TREND (Transparent Reporting of Evaluations with Nonrandomized Designs) guidelines for quasi-experimental studies.

### Ethical Considerations

#### Ethics Approval

This study was reviewed and approved by the ethics committee for Pharmacological Research (CEIm) of IISPV on October 31, 2024, meeting number 009/2024 (CEIm Code 159/2024). The study was conducted in accordance with the principles of the Declaration of Helsinki and Good Clinical Practice guidelines. Data confidentiality was protected in accordance with the Spanish Organic Law on the Protection of Personal Data and Guarantee of Digital Rights (03/2018, December 5). The study was prospectively registered at ClinicalTrials.gov (NCT06775509) on January 8, 2025, prior to the enrollment of the first participant, which took place on January 13, 2025.

#### Informed Consent

Written informed consent was obtained from all adult participants prior to enrollment. For participants aged <18 years, written informed consent was obtained from their parents or legal guardians, and age-appropriate written assent was obtained from the minor participants themselves, in accordance with the approved patient information sheet (H.I.P. v.1, September 27, 2023). All participants were informed that their participation was anonymous, confidential, and voluntary and that they retained the right to withdraw at any time without consequence. This study included children aged 8 to 17 years, considered a vulnerable population; to ensure adequate protection, parents or legal guardians were present at all study visits to provide support, ensure compliance with overnight contact lens use, and monitor for any adverse effects.

#### Privacy and Confidentiality

All participant data are treated with strict confidentiality. Anonymous identification codes were assigned to participants, and no personally identifiable information was included in the study dataset. Data are stored securely, and only the research team has access to the data, in compliance with applicable data protection regulations.

#### Participant Compensation

No financial compensation was offered to participants or their families.

## Results

The protocol received a favorable ethics opinion from the CEIm of IISPV in October 2024 (CEIm Code 159/2024). The study was prospectively registered at ClinicalTrials.gov (NCT06775509) on January 8, 2025, prior to the enrollment of the first participant on January 13, 2025.

Recruitment was completed. Data collection has been finalized, and data analysis is currently underway. The main study results are expected to be submitted for publication in January 2027. This study received no external funding.

The forthcoming analysis will characterize the change in anterior corneal spherical aberration after 1 month of treatment, together with changes in TCRP, the Q value, and epithelial thickness. Collectively, these parameters are expected to generate clinically relevant insights that may contribute to optimizing treatment strategies for myopia management and control and support more precise, evidence-based management approaches in contemporary ophthalmic practice.

## Discussion

### Principal Findings

This protocol manuscript outlines the methodological framework for evaluating the early optical quality changes induced by orthokeratology. The expected findings are consistent with the existing body of evidence on the optical effects of overnight orthokeratology. After 1 month of orthokeratology lens wear, an increase in anterior corneal spherical aberration is anticipated, reflecting the characteristic shift from a prolate to an oblate corneal profile induced by the central flattening and midperipheral steepening associated with this treatment. Concurrently, the study will assess changes in TCRP, Q value, and epithelial thickness distribution to determine whether they align with the known biomechanical and refractive effects of corneal reshaping therapy.

### Comparison to Prior Work

The proposed protocol aims to generate data that will enable direct comparison with previously published evidence. Hiraoka et al [7] reported a significant increase in higher-order corneal aberrations, particularly spherical aberration, following overnight orthokeratology, with these changes correlating with the degree of myopic correction. Similarly, Sun et al [6] demonstrated that overnight orthokeratology induces significant changes in corneal surface shape and optical quality, with the greatest optical degradation occurring within the first month of treatment. Lian et al [11] further documented that corneal reshaping and wavefront aberrations evolve rapidly during the initial weeks of orthokeratology wear, stabilizing thereafter. With respect to epithelial changes, Kim et al [12] and Alharbi and Swarbrick [13] reported nonuniform epithelial thickness redistribution following orthokeratology, with central thinning and midperipheral thickening consistent with the refractive changes observed. The present study contributes objective, tomographic, and OCT-based evidence to this body of work, providing novel insights into the relationship between epithelial redistribution, corneal asphericity, and induced aberrations in a pediatric and young adult population.

### Strengths and Limitations

Although numerous systematic reviews and meta-analyses on the optical effects of orthokeratology have been published in recent years, including studies examining the influence of back optic zone diameter on corneal HOAs and myopia control, the existing evidence is predominantly derived from studies using conventional videokeratography or wavefront aberrometry of the total ocular wavefront, and most previous reports have characterized aberrations and corneal power based on the anterior corneal surface alone. A strength of the present study lies in its systematic evaluation of early whole-cornea optical changes using Pentacam tomography and anterior segment OCT, instruments that allow simultaneous assessment of corneal aberrations derived from both the anterior and posterior corneal surfaces, TCRP derived from both corneal surfaces rather than the anterior surface alone, the Q value, and epithelial thickness distribution within the first month of treatment. This multiparameter tomographic approach, applied within a standardized measurement protocol, with all measurements performed in the morning, 1 to 3 hours after lens removal, in a pediatric and young adult population (aged 8-21 years) with parental oversight at all visits, aims to contribute objective and reproducible evidence that complements and extends the existing literature on the early optical effects of orthokeratology.

The quasi-experimental pre-post design lacks a concurrent control group. Consequently, observed changes cannot be attributed exclusively to the intervention, as confounding factors such as physiological corneal variation over time or regression to the mean cannot be fully excluded. The relatively short follow-up period of 1 month, while sufficient to capture early optical changes, does not allow the assessment of long-term stability or reversibility of the observed effects. Additionally, the study cohort presents inherent heterogeneity due to the wide age range of participants (aged 8-21 years). Although we acknowledge that corneal biomechanics and epithelial remodeling rates may differ across developmental stages, all participants will be analyzed as a single cohort without age-based stratification. We maintain this approach because the primary outcome—anterior corneal spherical aberration—is predominantly determined by the magnitude of myopic correction and lens design parameters rather than age. To our knowledge, no previous study has specifically demonstrated age-dependent variation in epithelial remodeling or aberrometric response within this age range. Although age is reported as a descriptive variable, future research with larger sample sizes should incorporate age-stratified analyses to formally explore potential variations in epithelial remodeling dynamics across developmental stages.

Accordingly, the findings should be interpreted as a pooled population-level effect across a heterogeneous age range rather than as an age-specific estimate. An adequately powered age-stratified analysis would require approximately 25 to 30 participants per subgroup, corresponding to a total sample of 50 to 60 participants, and remains a clear priority for future studies.

Cycloplegic refraction was performed at the baseline visit only to confirm eligibility and to inform orthokeratology lens design and was not repeated at follow-up. Consequently, changes in the components of refractive error cannot be assessed. Refraction measured over a cornea recently reshaped by orthokeratology is of limited reliability, and repeated cycloplegia in a pediatric population entails additional burden; nevertheless, future studies incorporating repeated refraction, where clinically appropriate, would allow this question to be addressed.

Sleeping position was not objectively monitored. Parents and guardians were advised to encourage a supine sleeping position as part of routine clinical counseling, but adherence to this recommendation was neither verified nor recorded. As sleeping position may influence lens centration, future studies should incorporate objective monitoring, for example, by means of positional sensors.

### Future Directions

The present study represents a foundational step in characterizing the early optical effects of orthokeratology in a clinical setting. Future research should include longer follow-up periods to assess the temporal evolution and stability of corneal aberrations beyond the first month of treatment, as well as include a control group to allow causal attribution of the observed changes. Ultimately, multicenter randomized controlled trials with extended follow-up would strengthen the evidence base and support more precise clinical recommendations for orthokeratology use in myopia management.

### Dissemination Plan

The results of this study will be submitted for publication in a peer-reviewed scientific journal in January 2027. Findings will be reported in accordance with the TREND reporting guidelines for quasi-experimental studies.

### Conclusions

One month of overnight orthokeratology is anticipated to produce measurable changes in anterior corneal spherical aberration, TCRP, Q value, and epithelial thickness distribution in patients with myopia aged 8 to 21 years. These changes align with the known optical effects of corneal reshaping therapy, confirming the early onset of aberrometric modifications within the first month of treatment. The systematic and standardized assessment of these parameters using Pentacam tomography and anterior segment OCT is expected to provide objective, reproducible evidence that may assist clinicians in counseling patients on the optical implications of orthokeratology wear. Future studies with longer follow-up periods and controlled designs are required to establish the long-term stability of these changes and to further optimize orthokeratology as a myopia control strategy.

## Supplementary material

10.2196/94476 Checklist 1 TREND checklist.

## Abbreviations

CEIm: *Comité de Ética de la Investigación con medicamentos*
FDA: Food and Drug Administration
GEE: generalized estimating equations
HOA: higher-order aberration
IISPV: *Institut d'Investigació Sanitària Pere Virgili*
MAR: missing at random
MPMVA: maximum plus for maximum visual acuity
OCT: optical coherence tomography
RGP: rigid gas-permeable
RMS: root mean square
Sim-K: simulated keratometry
TCRP: total corneal refractive power
TREND: Transparent Reporting of Evaluations with Nonrandomized Designs
