# Supplementary material for: Optical Quality of the Cornea After Orthokeratology Based on Total Corneal Refractive Power, Corneal Asphericity, and Epithelial Thickness: Protocol for a Prospective Quasi-Experimental Pre-Post Study
Source: JMIR Res Protoc. 2026 Aug 12;15:e94476. doi: 10.2196/94476 (PMC13464486; doi:10.2196/94476)
Supplement: Supplementary file 1 [file resprot-v15-e94476-s001.pdf]

TREND Checklist — Final Version

Transparent Reporting of Evaluations with Nonrandomized Designs

Manuscript: Optical quality of the cornea in orthokeratology: influence of total corneal refractive power, corneal asphericity, and epithelial thickness. Study protocol.

Study design: Prospective quasi-experimental pre-post study | Unit of analysis: eye (23 patients, 43 eyes)

Trial Registration: ClinicalTrials.gov NCT06775509

Ethics: CEIm IISPV, Code CEIM 159/2024, approved 31 October 2024

Data collection: December 2024 – January 2026 | Expected publication: January 2027

Status legend

✅Covered — item fully addressed in the manuscript

N/A — not applicable to this study design (single-arm pre-post, no randomization or control group)

Item #	Section	Descriptor	How addressed in manuscript	Status	Location in manuscript
1	Title and Abstract	Title and abstract	Title clearly states study design and topic. Abstract includes all structured sections (Background, Objectives, Methods, Results, Conclusions) within the 450-word limit.	✅ **Covered**	Title page / Abstract
2	Introduction	Scientific background	Background explains the corneal changes induced by Ortho-K and the rationale for studying optical quality including spherical aberration. Introduction ends with a clear transition to study objectives.	✅ **Covered**	Introduction
3	Introduction	Study objectives	Primary objective: change in anterior corneal spherical aberration (C[4,0], μm) after 1 month. Secondary objectives: TCRP (3/5/7 mm), Q value, epithelial thickness. Cycloplegic refraction used at baseline only for eligibility and lens design.	✅ **Covered**	Methods – Study objectives
4	Methods	Participants – eligibility criteria	Inclusion: age 8–21 years, myopia −1.00 to −5.00 D, astigmatism ≤1.50 D following-the-rule, BCVA 20/20, lens centration <0.5 mm. Exclusions: prior refractive procedures, corneal pathology, dry eye, glaucoma, retinal pathology, strabismus, ptosis, amblyopia, ocular allergy or infection.	✅ **Covered**	Methods – Participant selection
5	Methods	Settings	Clínica Oftalmològica Licari Visión, Tarragona, Spain (private ophthalmology clinic).	✅ **Covered**	Methods – Study design and setting
6	Methods	Intervention	Paragon CRT Ortho-K lenses (Paragon Vision Sciences, Mesa, AZ, USA), 8–10 hours/night for 1 month. Fitting: fluorescein pattern + topographic centration (Pentacam axial/tangential maps). Adherence: questionnaire, daily diary, parental report; minimum 8 hours/night. Discontinuation criteria: decentration >0.5 mm, infection, intolerance, adverse event, voluntary withdrawal. Adverse events recorded in clinical record and reported to CEIm.	✅ **Covered**	Methods – Description of the intervention

Item #	Section	Descriptor	How addressed in manuscript	Status	Location in manuscript
7	Methods	Objectives and hypotheses	Primary outcome: change in anterior corneal spherical aberration (Zernike C[4,0], µm) after 1 month. Secondary outcomes: TCRP, Q value, epithelial thickness. All measurements standardized: morning, 1–3 hours after lens removal.	✓ Covered	Methods – Study objectives / Study variables
8	Methods	Outcomes	Corneal aberrations: Pentacam (Zernike coefficients, 3/5/7 mm pupils; both anterior and posterior corneal surfaces). Epithelial thickness: REVO SOCT 27 OCT (Optopol, automatic segmentation). TCRP: Pentacam (both corneal surfaces, refractive index 1.376, 3/5/7 mm). Pupillometry: MYAH (Topcon, photopic/mesopic/scotopic). Axial length: MYAH (descriptive only). Cycloplegic refraction: cyclopentolate 1% x2 drops (5 min apart), refraction at 45 min; autorefractometry + retinoscopy + subjective MPMVA; baseline only.	✓ Covered	Methods – Study variables and data sources
9	Methods	Sample size	Optical reference threshold used for sample size estimation: 0.14 µm (Rayleigh criterion, λ/4 = 0.555/4); not a validated clinical threshold of visual quality. α=0.05, power=95% (β=0.05), SD=0.166 µm → 19 participants minimum. With 20% dropout: 23 participants. Calculated with GRANMO v.8.	✓ Covered	Methods – Sample size
10	Methods	Assignment of intervention	Single-arm quasi-experimental pre-post study. No randomization. All eligible participants received the intervention. No control group.	✓ Covered	Methods – Study design
11	Methods	Blinding	Not applicable — single-arm pre-post study with no control group.	N/A	Not applicable
12	Methods	Unit of allocation	Unit of analysis: eye. Both eyes of each participant included (23 patients, 43 eyes). Inter-eye correlation accounted for using Wilcoxon signed-rank test with clustering adjustment at the patient level (SPSS).	✓ Covered	Methods – Statistical Analysis
13	Methods	Statistical methods	Descriptive statistics: mean/SD (normal data); median/IQR (non-normal). Normality: Kolmogorov-Smirnov. Pre-post comparisons: Wilcoxon signed-rank with clustering adjustment for bilateral data (SPSS). Significance p<0.05. Multivariate regression not performed (n=23/43 eyes insufficient to avoid overfitting). Follow-up visits at 1 night and 1 week are safety monitoring only; primary analysis is baseline vs 1 month.	✓ Covered	Methods – Statistical Analysis
14	Results	Participant flow	Data collection commenced December 2024; recruitment completed with 23 participants enrolled (43 eyes). Study currently in data-processing phase; primary analysis underway. Results expected January 2027.	✓ Covered	Abstract – Results
15	Results	Recruitment	Recruitment period: December 2024 – January 2026. 23 participants enrolled.	✓ Covered	Abstract – Results
16	Results	Baseline data	Baseline characteristics (age, sex, refraction, corneal parameters) to be reported in results paper. Personal variables: age, sex. Ocular variables: epithelial thickness, corneal power, myopia/astigmatism, pupil diameter, axial length.	✓ Covered	Methods – Study variables
17	Results	Baseline equivalence	Not applicable — single-arm study with no comparison group.	N/A	Not applicable

Item #	Section	Descriptor	How addressed in manuscript	Status	Location in manuscript
18	Results	Numbers analyzed	23 patients, 43 eyes. Both eyes analyzed with clustering adjustment for inter-eye correlation. Results to be submitted for publication in January 2027.	✓ **Covered**	Methods – Statistical Analysis / Abstract Results
19	Results	Outcomes and estimation	Primary: anterior corneal spherical aberration change (μm). Secondary: TCRP, Q value, epithelial thickness. Results to be reported in results paper (submission January 2027).	✓ **Covered**	Methods – Study variables
20	Results	Ancillary analyses	Ancillary analyses to be reported in results paper.	✓ **Covered**	Methods – Statistical Analysis
21	Results	Adverse events	No serious adverse events recorded. Adverse event monitoring: recorded in clinical records and reported to CEIm (Code CEIM 159/2024).	✓ **Covered**	Methods – Description of the intervention
22	Discussion	Interpretation, generalizability, limitations	Limitations: quasi-experimental pre-post design without control group; wide age range (8–21 years) without subgroup analysis; short follow-up (1 month). Strengths: multi-parameter tomographic approach (Pentacam + OCT); whole-corneal assessment of aberrations and refractive power (anterior and posterior surfaces), rather than the anterior surface alone as in most prior reports; standardized measurement timing; paediatric population with parental oversight. Dissemination: peer-reviewed journal, January 2027, following TREND guidelines.	✓ **Covered**	Discussion – Strengths and Limitations / Dissemination Plan

All 22 TREND items are either fully covered or declared not applicable to this study design. No pending items remain.
